# A Systematic Review Protocol to Assess the Effects of Physical Activity on Health and Quality of Life Outcomes in Adolescent Cancer Survivors

**DOI:** 10.2196/resprot.5383

**Published:** 2016-03-30

**Authors:** Amanda Wurz, Jennifer Brunet

**Affiliations:** ^1^ University of Ottawa Faculty of Health Sciences School of Human Kinetics Ottawa, ON Canada

**Keywords:** controlled clinical trial, randomized controlled trial, adolescent, oncology, neoplasm, exercise, quality of life.

## Abstract

**Background:**

The benefits of physical activity for child and adult cancer survivors have been summarized in previous systematic reviews. However, no review has summarized the evidence for adolescent cancer survivors.

**Objective:**

This paper describes the design of a protocol to conduct a systematic review of published studies examining the effects of physical activity on health and quality of life outcomes for adolescent cancer survivors.

**Methods:**

Several guidelines informed the development of this protocol. The Preferred Reporting Items for Systematic Review and Meta-Analysis Protocols guidelines provided the structure by which to conduct and report the protocol; though some adaptations were made with regards to search terms, data synthesis, and evaluating the risk of bias. The Cochrane Handbook for Systematic Reviews of Interventions was used to guide research question development, search term selection, and the data extraction form. The Consolidated Standards of Reporting Trials guidelines helped inform the data extraction form. Lastly, the Guidance on the Conduct of Narrative Synthesis in Systematic Reviews informed the data synthesis. Ten electronic databases were identified and a search strategy was developed using a combination of Medical Subject Headings terms and keywords that were developed by the authors and peer reviewed by a university librarian. Both authors independently screened eligible studies for final inclusion, and data were abstracted using a form developed by the research team. A decision was made to synthesize all data narratively.

**Results:**

The review has now been completed, peer-reviewed, and accepted for publication in a forthcoming issue of JMIR Cancer.

**Conclusions:**

As this will be the first systematic review on this topic, outlining the protocol ensures transparency for the completed review. Further, this protocol illustrates how elements from several guidelines were incorporated to answer the research question (ie, what is the effect of physical activity on health and quality of life outcomes in adolescent cancer survivors). This flexible approach was necessary as a function of the paucity of available research on this topic.

## Introduction

Over 7500 adolescents (defined herein as individuals between 13 to 19 years of age) living in North America are diagnosed with cancer each year [1,2] and become a cancer survivor. The National Cancer Institute defines a cancer survivor from the point of diagnosis onward [3]. As such, a cancer survivor may be actively receiving treatment (ie, on-treatment) or have completed treatment (ie, off-treatment). This definition of a cancer survivor will be used throughout this paper. Approximately 80% will survive but will be at an increased risk for disability, morbidity, and mortality [4-8]; impaired physical, psychological, and social functioning [9,10]; and reduced quality of life [11,12]. In addition, normative growth, maturation, and development may be disrupted because of the disease and its treatments [13]. The National Comprehensive Cancer Network and the Institute of Medicine have identified adolescents and young adults as a distinct group of survivors who need to come to the forefront of efforts to lessen the impact of cancer [14,15]. Therefore, many researchers are taking action to identify complimentary therapies that can reduce the side effects of cancer and conventional cancer treatments.

Physical activity has been suggested as an effective adjunctive therapy to minimize the disruptions caused by cancer and its treatments [16]. Several reviews are available reporting on the safety and effects of physical activity on physical, psychological/emotional, and social health for child and adult cancer survivors [17-22]. However, no review has focused exclusively on adolescent cancer survivors despite acknowledgment that they are in a distinct developmental stage during their cancer experience [23]. In an effort to fill this gap, a review summarizing this information is needed. Thus, a systematic review protocol was developed. Both authors adhered to this protocol to comprehensively review and synthesize published studies focusing on the effectiveness of physical activity for promoting health and quality of life among adolescent cancer survivors. In doing so, adaptations from existing guidelines for systematic review protocols and systematic reviews (ie, Preferred Reporting Items for Systematic Review and Meta-Analysis Protocols, Cochrane Handbook for Systematic Reviews of Interventions, Consolidated Standards of Reporting Trials, Guidance on the Conduct of Narrative Synthesis in Systematic Reviews) were necessary because of the challenges of developing and conducting a systematic review on a topic with a paucity of available research. Therefore, the aim of this systematic review protocol was to outline the step-by-step process underlying the design and conduct of a systematic review exploring the effectiveness of physical activity for promoting health and quality of life in adolescent cancer survivors.

## Methods

This review protocol followed the Preferred Reporting Items for Systematic Review and Meta-Analysis Protocols guidelines [24]. The guidelines consist of 17 items to facilitate the preparation and reporting of systematic review and meta-analysis protocols. Items cover 3 aspects of reporting: (1) administrative information (5 items), (2) introduction (2 items), and (3) methods (10 items). Specifically, adhering to Preferred Reporting Items for Systematic Review and Meta-Analysis Protocols guidelines from study inception promoted systematically defined concepts and inclusion criteria, provided a decision analytic framework, and offered guidance for writing up the final protocol. In addition, elements from the Cochrane Handbook for Systematic Reviews of Interventions, the Consolidated Standards of Reporting Trials, and the Guidance on the Conduct of Narrative Synthesis in Systematic Reviews were used as applicable. Each of these guidelines and the necessary changes (and the rationale for such changes) are provided below.

### Inclusion Criteria

Studies were included if they were published in English in peer-reviewed scientific journals. The types of participants, interventions, outcomes, and studies that were considered for inclusion are described below.

#### Types of Participants

Studies were included if participants were adolescents aged 13 to 19 years who were diagnosed with any type of cancer and were at any point on the cancer trajectory (ie, diagnosis, treatment, post-treatment, palliation). Careful consideration was given in selecting this age range. Although there are several definitions currently being used, the ultimate goal of this review was to determine the effectiveness of physical activity for cancer survivors in their teenage years (ie, 13 to 19 years). Also, the definition selected adheres with recent efforts by other research groups [10]. Further, the decision to include cancer survivors on- and off-treatment is consistent with the National Cancer Institute’s definition of a cancer survivor [3]. In cases where a wider age range was used, 50% or more of the sample had to contain participants meeting the above age criteria. This decision was made to increase the number of studies eligible for inclusion.

#### Types of Interventions

Physical activity was defined as consisting of aerobic training, resistance training, flexibility training, combined training, or any other form of physical movement with the goal of increasing energy expenditure. Physical activity interventions were limited to those that included more than one session. No restrictions were placed on where the intervention was delivered (eg, hospital, home, school, community), the format of the intervention (eg, group-based, individual), or the individual delivering it (eg, physiotherapist(s), exercise professional(s), researcher(s), personal trainer(s), parent(s)).

#### Types of Outcome Measures

Studies that included measures of health and/or quality of life as primary or secondary endpoints were included. Health outcomes included any participant-reported or objective assessment of physiological functioning (eg, fatigue, body composition, cardiovascular capacity, strength, flexibility). Quality of life outcomes included any participant-reported assessment of functioning across physical, psychological/emotional, and social domains.

#### Types of Studies

Only experimental study designs were included. Randomized controlled trials or controlled clinical trials were selected, as these study designs constitute the most robust form of clinical evidence [25]. Further, there had to be at least pre-post assessments. This decision was made so that any change observed over the course of the intervention could be more confidently attributed to the physical activity intervention [26].

### Exclusion Criteria

Interventions that had multiple program features (eg, multiple behavior change strategies, nutrition counseling) were excluded, as any observed effects as a result of the intervention could not be attributed solely to physical activity. Furthermore, those with insufficient details on the target population, intervention, comparison condition, or outcomes (after study authors were contacted and it was determined the requested information was unavailable) were ineligible.

### Data Sources and Search Strategy

A search strategy was developed using an iterative process based on recommendations from a university librarian (YL) and the methods sections (ie, keywords, procedures) from existing reviews on physical activity and cancer [18,20]. The strategy included a combination of Medical Subject Headings terms and keywords related to the population (eg, adolescent, young adult, young person, teenager, cancer, neoplasm), intervention (eg, exercise, physical fitness, aerobic exercise, resistance training, flexibility), comparison condition (eg, control groups, usual care), and outcomes (eg, health-related fitness, range of motion, quality of life, mood). During a preliminary search of MEDLINE, a limited number of studies were identified. Therefore, the search strategy was revised by excluding terms related to outcomes in order to reduce the likelihood of limiting the search to predefined outcomes and to maximize the number of studies retrieved (see Appendix 1 for the final MEDLINE search strategy). The revised search strategy was then translated and the following 10 electronic databases were searched from inception to November 2015: CINAHL, Cochrane Central Register of Controlled Trials, Embase, LILACS, MEDLINE, PEDro, Physical Education Index, PsycINFO, PubMed, and SPORTDiscus. Similar to other systematic reviews [17-21], following this, the reference lists of all studies meeting the inclusion criteria and any relevant reviews identified during the electronic database search were scanned to identify additional studies.

#### Study Selection

All studies identified in the database search were exported to a reference managing software [27] and duplicate records were deleted. Both authors independently reviewed the titles and abstracts of all references. Articles clearly not meeting the established inclusion/exclusion criteria were excluded. Following this, both authors independently screened the full text articles of abstracts identified to select the studies to be included. Then the reference lists of included studies and relevant reviews were scanned to identify additional studies. Both authors independently screened the full texts of these additional articles to determine inclusion/exclusion. Third-party arbitration (AJ and CO) was available to resolve any inconsistencies in the selection of studies for inclusion/exclusion. A Preferred Reporting Items for Systematic Review and Meta-Analysis flow diagram [28,29] was prepared to show the overall process of study selection and the number of citations reviewed at each stage of this review.

#### Data Collection

A data extraction tool was developed specifically for this review based on recommendations provided in the Cochrane Handbook of Systematic Reviews of Interventions [30]. In cases where details were missing on study design, population, intervention, or outcomes, the authors of included studies were contacted by email. After the first contact attempt, if no response was received, the study authors were contacted 2 more times approximately 3 to 4 weeks apart. The following information was extracted from each included article: (1) sources of data, (2) study design and study period, (3) characteristics of the population (ie, number of participants randomized, age, type(s) of cancer diagnosed, cancer phase), (4) intervention characteristics (ie, supervision, setting, length, frequency, intensity, activity type(s)), (5) outcome measures (ie, health and/or quality of life), and (6) outcomes (ie, health and/or quality of life).

In addition to extracting the above standard data, additional information was documented on the use of theoretical frameworks (ie, whether the study was informed by theory). The use of intention-to-treat analysis was also recorded since intention-to-treat analyses generally provide an unbiased estimate of treatment effect. Intention-to-treat is a well-regarded approach to the design, conduct, and analysis of a trial [31], and it is a key component in the Consolidated Standards of Reporting Trials guidelines [32]. Additionally, data on variables not considered to be health and/or quality of life outcomes (eg, intervention acceptance, adverse events, adherence to the study protocol) were extracted to provide a more comprehensive understanding of the state of the literature.

### Data Synthesis

Given that the main purpose of the systematic review was to comprehensively review and synthesize published studies focusing on the effectiveness of physical activity for promoting health and quality of life among adolescent cancer survivors, all data extracted from the articles were presented narratively in text and summary tables. This decision was made because narrative synthesis provides a broad overview of relevant information through a textual approach and is appropriate when it is expected that studies will be too heterogeneous to allow for a quantitative summary [33]. Heterogeneity of studies was assessed according to content, rather than by performing statistical tests for homogeneity. It was expected that the studies included in this review would vary widely. This was based on a recent review conducted with pediatric cancer survivors that found large variability across studies [17]. To ensure the quality of the narrative synthesis, the Guidance on the Conduct of Narrative Synthesis in Systematic Reviews was followed as appropriate to accurately report the review search results and analysis summary [33]. Specifically, the included studies were carefully reviewed and the limitations of each (ie, quality assessment) were described. Additionally, the entire data extraction and synthesis process was carefully detailed, and objective third-party review (AJ) was utilized.

## Results

The review has now been completed, peer-reviewed, and accepted for publication in a forthcoming issue of JMIR Cancer [34]. 

## Discussion

Whereas other recent systematic reviews were undertaken to investigate the benefits of physical activity for children and adults diagnosed with cancer [17-20], none have focused exclusively on adolescents. Further, few, if any, have published a detailed protocol either independently or as supplemental material. Thus, this protocol adds to the field of physical activity and cancer. A key strength of this review protocol is the use of multiple gold standard guidelines. By incorporating different elements from each guideline, a solid framework and structure was created by which the research question could be answered. Additional strengths are the inclusion of a university librarian (YL) with experience conducting systematic reviews who assisted with peer-reviewing the search strategy, the application of a data extraction template, and a flexible approach to data acquisition and synthesis.

Notwithstanding the strengths, there were key challenges to preparing and finalizing this review protocol. First, there were challenges formulating the inclusion/exclusion criteria. In general, given the complexity and breadth of definitions for adolescent cancer survivors and the numerous iterations of physical activity interventions, careful consideration was given to ensuring the best evidence was identified to answer the research question. Second, identifying pertinent literature was difficult given the paucity of results obtained in preliminary tests of the search strategy. A flexible approach to search terms and keywords was necessary to ensure more studies were identified for review. Third, in light of the lack of research focused on adolescent cancer survivors, synthesizing and interpreting data was assumed to be a challenge. Thus, a narrative approach to data synthesis was selected. New topics pose inherent challenges for systematic review protocol development. As the literature in this area grows, so will the opportunities to refine protocols, re-run searches, and update findings. However, until then, protocols should be disseminated to ensure transparency of completed reviews and aid other researchers in developing their review protocols.

## Appendix

Multimedia Appendix 1 MEDLINE search strategy.
