# Supplementary material for: A Systematic Review Protocol to Assess the Effects of Physical Activity on Health and Quality of Life Outcomes in Adolescent Cancer Survivors
Source: JMIR Res Protoc. 2016 Mar 30;5(1):e54. doi: 10.2196/resprot.5383 (PMC4830903; doi:10.2196/resprot.5383)
Supplement: Multimedia Appendix 1 [file resprot_v5i1e54_app1.pdf]

1. exp Exercise/

2. Physical Fitness/

3. Motor Activity/

4. (physical adj2 (activit* or exercis* or train* or fitness)).ti,ab.

5. aerobic*.ti,ab.

6. endurance.ti,ab.

7. (strength adj1 (activit* or exercis* or train* or fitness)).ti,ab.

8. flexibility.ti,ab.

9. (resistance adj1 (activit* or exercis* or train* or fitness)).ti,ab.

10. (musc* adj2 (exercis* or strength)).ti,ab.

11. stretch*.ti,ab.

12. fitness.ti,ab.

13. movement.ti,ab.

14. (physical adj2 therap*).ti,ab.

15. (physical adj2 rehab*).ti,ab.

16. yoga.ti,ab.

17. or/1-16

18. exp neoplasms/

19. (leuk?emia or carcinoma or adenocarcinoma or lymphoma).ti,ab.

20. (cancer* or oncolog* or tumo?r).ti,ab.

21. or/18-20

22. Adolescent/

23. young*.ti,ab.

24. (young adj1 person).ti,ab.

25. (teen* or youth or adolescen* or p?ediatr*).ti,ab.

26. or/22-25

27. patients/ or survivors/

28. (patient or surviv*).ti,ab.

29. (inpatient* or outpatient*).ti,ab.

30. inpatients/ or outpatients/

31. or/27-30

32. 21 and 26 and 31

33. Control Groups/

34. (control adj1 (group* or condition*)).ti,ab.

35. (usual adj1 (care or treat*)).ti,ab.

36. (standard adj1 (care or treat*)).ti,ab.

37. (comparison adj1 (group* or condition*)).ti,ab.

38. (comparison* or control*).ti,ab.

39. or/33-38

40. 17 and 32 and 39
